# Strategies for implementing HIV self-testing in diverse populations: an integrative review

**DOI:** 10.1590/0034-7167-2024-0597

**Published:** 2025-12-12

**Authors:** Maiara Bezerra Dantas, Gilmara Holanda da Cunha, Régia Christina Moura Barbosa Castro, Maria Elisa Curado Gomes, Ane Kelly Lima Ramalho, Vanessa Sousa dos Santos

**Affiliations:** I Universidade Federal do Ceará. Fortaleza, Ceará, Brazil

**Keywords:** Self-Testing, HIV, Diagnosis, Health Promotion, Disease Prevention., Autoevaluación, VIH, Diagnóstico, Promoción de la Salud, Prevención de Enfermedades.

## Abstract

**Objectives:**

to identify strategies for implementing HIV self-testing in populations.

**Methods:**

an integrative review of MEDLINE/PubMed, Scopus, CINAHL, Web of Science, and LILACS databases. Full articles available online, in Portuguese, English, Spanish, or French, regardless of the year of publication, were included. Letters to the editor, review articles, and duplicates, rapid tests performed by professionals, and home self-tests sent to a laboratory without on-site results, were excluded. The sample of 24 articles had their level of evidence assessed using descriptive data analysis.

**Results:**

the most prevalent strategy for implementing self-testing was community distribution through home visits, mail, and distribution points such as bars, nightclubs, and transportation terminals. Peer distribution was significant among men who have sex with men, young people, and trans women, with primary delivery directly to the individual or secondary delivery, where one individual delivers to others. Social and technological media facilitated patient recruitment, delivery, and connection to healthcare services.

**Conclusions:**

the distribution of HIV self-tests in the community was the most accessible strategy, which increased the use of combination HIV prevention methods and initiation of antiretroviral therapy. The main challenges to implementing self-testing were financial costs and acceptance, due to fear of results, stigma, and intimate partner violence.

## RESUMEN

**Objetivos:**

identificar estrategias para implementar la autoprueba de VIH en poblaciones.

**Métodos:**

revisión integrativa de las bases de datos MEDLINE/PubMed, Scopus, CINAHL, *Web of Science* y LILACS. Se incluyeron artículos completos disponibles en línea, en portugués, inglés, español o francés, independientemente del año de publicación. Se excluyeron las cartas al editor, los artículos de revisión, los duplicados, las pruebas rápidas realizadas por profesionales y las pruebas de autoevaluación caseras enviadas a un laboratorio sin resultados in situ. El nivel de evidencia de la muestra de 24 artículos se evaluó mediante análisis descriptivo de datos.

**Resultados:**

la estrategia más frecuente para implementar el autoprueba fue la distribución comunitaria mediante visitas domiciliarias, correo y puntos de distribución como bares, discotecas y terminales de transporte. La distribución entre pares fue significativa entre hombres que tienen sexo con hombres, jóvenes y mujeres trans, con entrega directa a la persona o entrega secundaria, donde una persona distribuye a otras. Las redes sociales y las tecnologías facilitaron el reclutamiento, la entrega y la conexión de pacientes con los servicios de salud.

**Conclusiones:**

la distribución de autopruebas de VIH en la comunidad fue la estrategia más accesible, lo que incrementó el uso de métodos combinados de prevención del VIH y el inicio de la terapia antirretroviral. Los principales desafíos para implementar las autopruebas fueron los costos financieros y la aceptación de los pacientes, debido al miedo a los resultados, el estigma y la violencia de pareja.

## INTRODUCTION

The 95-95-95 target of the Joint United Nations Programme on HIV/AIDS recommends that, by 2030, 95% of people living with HIV (PLHIV) should be diagnosed, 95% on antiretroviral therapy (ART) and 95% in viral suppression^(1)^. Achieving these goals will enable the end of the AIDS pandemic as well as benefits for global health and the economy^(2)^. For this, testing is essential, as it is the first step in connecting PLHIV to healthcare.

In this context, self-testing can contribute to the early detection of HIV and the achievement of global control goals, as it is a practical strategy to improve access to testing^(3)^. HIV self-testing is convenient because it provides privacy and mitigates challenges such as stigma and lack of anonymity in healthcare services, allowing it to reach inaccessible populations^(4)^. Studies show its potential for preventing and treating HIV infection^(5-7)^. However, this type of testing still presents some obstacles, such as the low level of education of the people who carry it out, the thought that the test is not reliable, as it is not carried out by a healthcare professional, the lack of prior counseling, the fear of a positive result, the weakened link with the healthcare service, in addition to the issue of human rights and freedom, since the partner or employer cannot force self-test^(8,9)^.

The delivery method of HIV self-tests has an impact on access to testing and frequency of distribution^(10)^, but there is evidence that a combination of several strategies is necessary for its implementation^(11-13)^. As this is a relatively new testing modality in Brazil, it is important to know which strategies work best, who the target audience is and to what extent^(14,15)^, with a view to increasing its effectiveness and helping to achieve goals^(4)^.

In Brazil, HIV self-testing is in the implementation phase and still has little visibility, despite there being guidelines for its distribution, which are aimed at sexual partners or peers of people seeking Pre-Exposure Prophylaxis (PrEP), sexual partners of PLHIV or peers of people tested in healthcare services, and places of sociability of key populations^(15)^. Delivery guidelines are suggested, and if users need them, support is available by phone or through the website^(15)^.

Since 2019, Brazil’s public health system has begun distributing tests in 14 cities. However, in a Brazilian survey of men who have sex with men (MSM), 67% reported that they were unaware that self-testing was available before enrolling in the study^(16)^. Another study found that the acceptability of HIV self-testing among MSM was 47.3%^(17)^. A systematic review of foreign studies showed that, of a sample of 710 participants, 33.1% reported that HIV self-testing was the preferred method of testing^(3)^.

Given the above, it is necessary to understand the different strategies for implementing HIV self-testing so that nurses, other members of the multidisciplinary health team and those responsible for public policies can define what best suits each population, with the aim of enabling accessibility and effective distribution of this input.

## OBJECTIVES

To identify strategies for implementing HIV self-testing in different populations.

## METHODS

### Ethical aspects

The writings of the articles and copyrights were respected, without modification of the writings found for the benefit of the study now proposed by the authors.

### Study design

This is an integrative literature review, conducted in six stages: 1. Research question identification; 2. Literature search; 3. Study categorization; 4. Assessment of included studies; 5. Interpretation of results; 6. Review presentation^(18)^.

### Research question development and selection criteria

The research question was developed based on the acronym PICo (Population, Interest, and Context): Population (general population), Interest (care strategies), Context (implementation of HIV self-testing). Thus, the guiding question was formulated: what are the strategies for implementing HIV self-testing? Preferred Reporting Items for Systematic Reviews and Meta-Analyses items were followed^(19)^. Full articles available online in Portuguese, English, Spanish, or French, covering the topic, regardless of the year of publication, were included to obtain the largest possible number of articles. Letters to the editor, review articles, duplicate articles, rapid tests performed by healthcare professionals, and home self-tests that involved sending material to a laboratory without providing results on site were excluded.

### Sample definition

The articles were selected from five databases: Medical Literature Analysis and Retrieval System Online (MEDLINE)/PubMed, Scopus, Cumulative Index to Nursing and Allied Health Literature (CINAHL), Web of Science, and Latin American and Caribbean Literature in Health Sciences (LILACS). The survey took place from July to December 2023, using the descriptors in Portuguese (“HIV Infections” and “Self-Test”) and English (HIV Infections AND Self-Testing) from the Health Sciences Descriptors and Medical Subject Headings, in addition to the Boolean operator AND.

Searches were conducted on the Coordination for the Improvement of Higher Education Personnel Journals Portal, using the Internet Protocol coverage of the *Universidade Federal do Ceará*, to broaden the scope of indexed and available journals. For a broader search, a test was performed in each database using combinations of various descriptors to identify as many articles as possible. Table 1 shows these intersections.

**Table 1:** Description of database search strategies, Fortaleza, Ceará, Brazil, 2024

Databases	Crossings	Articles
MEDLINE/PubMed	“HIV Infections” AND Self-Testing	3,252
Scopus	“HIV Infections” AND Self-Testing	756
CINAHL	“HIV Infections” AND “Self-Testing”	301
Web of Science	“HIV Infections” AND Self-Testing	138
LILACS	“HIV Infections” AND Self-Testing	42

After searching the databases, the articles were exported and identified on the Rayyan^®^ digital platform, a software that facilitates the process of grouping articles for reading and identifying duplicates^(20)^. Initially, the titles and abstracts of the articles were read to assess whether they met the inclusion criteria for the integrative review. Subsequently, selected articles were read in full by two independent, masked reviewers, based on the study’s guiding question and inclusion and exclusion criteria. In cases of disagreement between the researchers, a third reviewer was available.

### Data collection

The collected data were organized into an adapted guide with information on reference, year of publication, country of study, objectives, methodological characteristics (study design, population, sample), results, and conclusions. A table was then created with care strategies for implementing HIV self-testing in different populations. The articles’ levels of evidence were classified as: I. Evidence from a systematic review or meta-analysis of randomized controlled trials or clinical guidelines based on systematic reviews of randomized controlled trials; II. Evidence from at least one randomized controlled trial; III. Evidence from clinical trials without randomization; IV. Evidence from cohort and case-control studies; V. Evidence from a systematic review of descriptive and qualitative studies; VI. Evidence from a descriptive or qualitative study; VII. Evidence from the opinion of authorities or reports of expert committees^(21)^. Assessment was carried out by peers in an open manner, in which three authors of the study participated, in cases where there was no consensus regarding the definition of the levels of evidence.

## RESULTS

**Figure 1:**
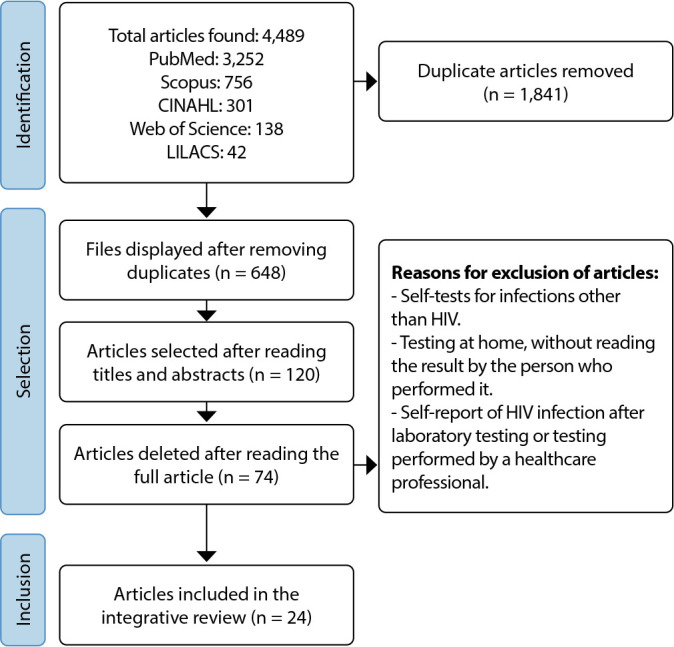
Flowchart of articles found and selected in the databases, Fortaleza, Ceará, Brazil, 2024

A total of 4,489 articles were selected, and 24 comprised the study. The year of publication of the articles ranged from 2017 to 2023. Regarding the levels of evidence, eight were level II^(22-29)^, one was level III^(30)^, three were level IV^(31-33)^, and 12 were level VI^(7,10,34-43)^. Figure 1 shows the selection flowchart.

The most prevalent strategies for self-test distribution in the studies were community distribution^(7,23-25,27,28,30,32,36,37,40,41)^ and peer distribution^(10,26,29,31,33,36,42)^. The most participating populations were MSM^(7,10,30,31,34,43)^, young people^(29,38-41)^ and trans women (TW)^(30,34)^. Chart 1 presents the descriptive synthesis of studies.

**Chart 1:** Strategies for implementing HIV self-testing in diverse populations, Fortaleza, Ceará, Brazil, 2024

Author/year/ country of production	Objectives	Method	Population	Results and conclusions	Level of evidence^(21)^
Edelstein *et al*. (2020) United States^(7)^	Describe the 2015-2018 Home Test Giveaway, its intervention process, and results.	Online cross-sectional study	n= 7,935	In online and home delivery, participants recommended HIV self-testing for privacy and convenience, reporting their results, and those who tested positive had a high connection with the healthcare service.	VI
Sekoni *et al*. (2022) Nigeria^(10)^	Assess the delivery of HIV self-testing to MSM by opinion leaders.	Qualitative study	12 distribution groups and 20 MSM	Opinion leaders described the successful delivery of HIV self-tests and that trust was a key factor. MSM viewed them as reliable sources of interpersonal education, with emphasis on self-testing training. Offering support, people management, patience, and perseverance are important characteristics of leaders, serving as a resource for promoting self-testing among MSM.	VI
Sande *et al*. (2018) Malawi^(22)^	Identify and quantify client costs of HIV self-testing services in rural communities in Malawi.	Cluster-randomized clinical trial Home survey and retrospective interview	n=749	For those who did incur a cost, the average was US$2.06. Age, location, time to purchase the self-test, visit to the institution, and place of residence influence costs. Of the participants, 23% had no cost to purchase a self-test. Participants who test in their own community have 61% lower financial costs, but the older age group has higher costs.	II
Neuman *et al*. (2021) Zambia^(23)^	Assess the impact of community-based HIV self-test distribution on testing services.	Cluster-randomized clinical trial	n=5,005	When compared to standard testing services in health facilities (control) and community-based distribution (intervention), only 58.0% of respondents in the intervention arm were aware of self-testing, compared with 28.3% in the control arm. Community-based distribution did not increase self-test use, but it did improve testing awareness.	II
Sibanda *et al*. (2021) Zimbabwe^(24)^	Assess the effect of encouraging bonding with the patient after HIV self-testing and the temporal trend towards initiation of ART.	Cluster-randomized clinical trial	n=7,146	There was no evidence of benefit in providing HIV self-test kit distributors with a linkage incentive beyond a fixed salary. Door-to-door distribution of HIV self-test kits in the community is feasible and has improved testing coverage.	II
Sibanda *et al*. (2021) Zimbabwe^(25)^	Compare a community-based model for HIV self-testing versus an established model in rural Zimbabwe.	Cluster-randomized clinical trial	n=11,150, Group 1: Community-led (n=5,683) Group 2: Paid distributor (n=5,467)	HIV self-testing uptake was lower in the community-led arm (21.6%) compared to the paid distribution arm (27.5%). Post-test engagement with healthcare services was higher in the community-led arm. Financial costs in the paid distribution arm were lower. The community-based intervention is promising. Costs tend to decrease over time and with greater implementation.	II
Sande *et al*. (2021) Malawi, South Africa, Zambia and Zimbabwe^(26)^	Assess the costs of adding HIV self-testing to standard HIV testing in four countries.	Cluster-randomized clinical trial	n=41,720	Counselors spent an average of 20-44% of their time on HIV self-testing activities. The cost in public facilities ranged from US$4.27 to US$13.42 per kit distributed across countries. Primary distribution ranged from US$4.27 to US$9.24, while secondary distribution ranged from US$6.46 to US$13.42.	II
Indravudh *et al*. (2021) Malawi^(27)^	Assess the effectiveness and safety of door-to-door distribution of HIV self-test kits in rural Malawi.	Randomized clinical trial	n=5,490	Door-to-door distribution of HIV self-tests by community distribution agents increased HIV testing (16.1%) and lifetime testing (6.3%), particularly among male adolescents aged 16 to 19.	II
Indravudh *et al*. (2021) Malawi^(28)^	Assess the costs and effects of community-led HIV self-testing delivery.	Pragmatic cluster-randomized trial	n=7,880	Community-led delivery demonstrated a low additional unit cost. Community-led HIV self-testing, combined with standard care, would not be cost-effective in settings with low HIV prevalence.	II
Shahmanes *et al*. (2021) South Africa^(29)^	Investigate two models of distribution of peer-based HIV self-testing for HIV prevention in young people.	Cluster-randomized clinical trial	n=24 peers	The distribution of HIV self-tests among peers reached a large number of young people. Social media-encouraged self-tests also reached a large number of young people, but resulted in fewer connections with healthcare services.	II
Rosadiño *et al*. (2023) Philippines^(30)^	Assess the feasibility of the HIV self-test distribution model in the community and assess its acceptability among MSM and TW.	Quasi-experimental study, with application of questionnaire	n=4,205	The implementation of self-testing with community distribution, carried out with the help of a virtual assistant and mailed tests, was feasible. Instructional materials and videos can help ensure proper use of the HIV self-test.	III
John *et al*. (2020) United States^(31)^	Determine the willingness of gay and bisexual men to offer HIV self-testing and ART to their partners, and to engage in notification, using the app.	Cohort study	n=786	When reporting to sexual partners, those who reported condomless anal sex with a casual partner were 3.21 times more likely to be willing to offer HIV self-testing and treatment for other sexually transmitted infections to their partners. Among single men, 93.8% of those who reported recent condomless anal sex with a casual partner were willing to offer HIV self-testing and treatment for other sexually transmitted infections to their partner.	IV
Driver *et al*. (2023) Kenya^(32)^	Assess whether completion of the self-test is associated with changes in sexual behavior and risk perception.	Cohort study, with questionnaire application	n=221	The community distribution models for self-tests included home distribution, pharmacies, and advertising in bars and nightclubs. After the HIV self-test, young people discussed testing more generally. Those who received the self-test in pharmacies and at home reduced unprotected sex. Offering self-tests to the population can encourage them to talk about testing with their partners.	IV
Gosset *et al*. (2023) France^(33)^	Assess the proportion and characteristics of people who distributed and received the self-test.	Prospective intervention study, with questionnaires	n=682	Self-tests were given to patients to distribute to people in their social networks. Those who distributed at least one self-test were more likely to have previously used the self-test. Older individuals were less likely to distribute the self-test. Prior awareness and training in its use improved distribution.	IV
Wirtz *et al*. (2017) Myanmar^(34)^	Investigate how HIV self-testing can benefit MSM and TW in post-test care.	Qualitative research, with focus group	n=35	Community education and mass media to inform MSM and TW about HIV self-testing are important to ensure MSM and TW access to instructions on self-test use.	VI
Durosinmi-Etti *et al*. (2021) Nigeria^(35)^	Understand facilities, barriers and communication needs for key populations in the provision of prep and HIV self-testing in Nigeria.	Cross-sectional study, with interviews and focus groups	n=1,169	Self-testing facilitators included face-to-face interaction, peer education sessions, social media, offering self-testing when purchasing a similar product, community networks, and messages in multiple languages. Face-to-face interaction was the most effective way to raise awareness about HIV self-testing (85% FSWs; 68% MSM), followed by social media (9.4% FSWs; 31.6% MSM). Peer influence is essential for spreading messages about HIV self-testing.	VI
Jamieson *et al*. (2021) South Africa^(36)^	Assess the impact of six HIV self-test kit distribution modalities in South Africa.	Cross-sectional telephone study and economic assessment	n=40,834	Secondary distribution to partners of patients on ART has an epidemiological impact, preventing new infections and improving survival, but it is a low-cost strategy. Taxi stand and workplace distribution are more cost-effective strategies.	VI
Matsimela *et al*. (2021) South Africa^(37)^	Analyze the cost, use, and relationship to aftercare of 11 HIV self-test kit distribution models.	Cross-sectional study using telephone survey	n=40,834	Average costs per HIV self-test kit ranged from US$4.87 (sex worker model) to US$18.07 (mobile integration model). The sex worker, transport terminal, and workplace model are more efficient and less costly. If the goal is to distribute kits cheaply and reach more HIV-positive individuals, secondary distribution by index case in facilities and through sex workers is more efficient.	VI
Nwaozuru *et al*. (2021) Nigeria^(38)^	Examine youth participation in marathon running to create youth-friendly healthcare services in Nigeria.	Descriptive study	n=48 young people	The three finalist approaches focused on decentralizing services to young people through mobile devices and tents, health technologies, or peer support services. The winning strategy was the creation of a mobile app for distributing and requesting HIV self-tests.	VI
Birdthistle *et al*. (2022) South Africa^(39)^	Assess the impact of the MTV series Shuga Down South on HIV prevention, acceptance, and awareness of HIV self-testing and prep among young people In South Africa.	Mixed study, online research	n=3,431	Those who watched the series had greater knowledge of HIV serology (71%), used HIV self-testing more (29%) or had a high interest in using it (83%), accessed testing services more frequently, and had more knowledge.	VI
Harrison *et al*. (2022) Malawi^(40)^	Explore barriers and facilitators of the link with healthcare services for HIV prevention and care after self-testing in young people.	Cross-sectional study, with interviews and focus groups	n=41	Communication about the benefits of testing and post-test referrals were poor, according to the young people. They valued encouragement from those they trusted, positive treatment experiences from others, and “strength of spirit”. Community distribution agents fostered trusting relationships and a greater understanding of the factors that prevented young people from connecting after self-testing.	VI
Aizobu *et al*. (2023) Nigeria^(41)^	Identify the facilitators and barriers to performing the self-test using the journey map methodology.	Exploratory study, with interviews and focus groups	n=80	Young people preferred that the pharmacy deliver HIV self-tests to their homes, as they were afraid of being seen in the pharmacy. Although they preferred this delivery model, they acknowledged the lack of counseling that they receive in public healthcare services, but not in pharmacies.	VI
Bulterys *et al*. (2023) Uganda^(42)^	Assess the perspectives of pregnant women and partners on the acceptability, barriers, facilitators, and strategies for HIV self-testing.	Exploratory study, with interviews and focus groups	n=122	Secondary distribution was acceptable, but couples preferred to test individually. Both were hesitant to test in the presence of their partner. Pregnant women reported the importance of receiving guidance on the use and interpretation of the self-test, as well as strategies for approaching their partner.	VI
Kwan *et al*. (2023) China^(43)^	Assess the implementation cascade of a social network-based HIV self-testing approach with MSM.	Cross-sectional study, with application of questionnaire	n=442	Using MSM social networks to recruit other men is a viable approach, but it requires motivated peers. Having confident users for recruitment can be beneficial, as they have experience, knowledge, and relationships with peers.	VI

*MSM - men who have sex with men; ART - antiretroviral therapy; TW - trans woman; PrEP - Pre-Exposure Prophylaxis; FSWs - female sex workers.*

The HIV self-test distribution can occur primarily, in which the self-test is delivered directly to individuals, within an institution or other location, or secondary, in which it is delivered to an individual so that they can then give it to another person, such as their sexual partner^(36)^ or peers^(31)^.

Community distribution was the most common^(7,23-25,27,28,30,32,36,37,40,41)^. This can happen in numerous ways, with the help of people called community distributors, who deliver self-tests through home visits^(23,32,41)^, through the post office^(7,30)^ and at distribution points in places frequented by the population to be reached, such as bars and nightclubs^(32,36)^. At transport terminals (taxi ranks and train stations with high pedestrian traffic), self-tests are offered to passengers, taxi drivers and street vendors.

At these terminals, distributors demonstrate the use of self-testing, offering them to interested parties for private use off-site^(36,37)^. Through door-to-door distribution, people go to homes to offer and deliver the self-test^(24,25,27)^. Distribution can also occur with and without financial incentives, where distributors receive payment to carry out this activity^(24,25)^.

A significant distribution among peers was observed in the studies assessed, occurring among sexual partners^(31)^, among pregnant women to their sexual partners during prenatal care^(26,42)^ and among people with HIV to their partners^(26,36)^, handing it over to people in their social networks^(29,33)^. One of the studies addressed the participation of opinion leaders, who were respected people in the community, to distribute HIV self-tests among MSM as well as encourage them to seek healthcare^(10)^.

To distribute self-tests, some researchers have used social media to facilitate the collection, delivery, and connection of patients to healthcare services after testing. Mobile applications^(31,38)^, such as dating apps^(7)^ or apps for notifying HIV-positive partners^(31)^, WhatsApp^®(10)^, media such as TV series^(39)^, and online advertisements in digital media, have been used^(7)^.

Regarding the acceptability of HIV self-testing, participants who self-tested reported high levels of health follow-up when testing positive and that they would recommend this form of testing due to privacy and home convenience^(7,34)^. Concerning the aspects that hinder the acceptability of HIV self-testing, there is the lack of pre-test and post-test counseling, as they can negatively impact mental health, with less possibility of revealing HIV+ status, notification and surveillance of cases^(34)^.

In a study of gay and bisexual men, the majority who were diagnosed through HIV self-testing considered it acceptable to provide their partner with treatment for other sexually transmitted infections^(31)^. Pregnant women and their partners preferred to do the self-test independently, i.e., not have it offered by their partner^(42)^.

Although acceptable for couples in relationships of trust and mutual understanding, pregnant women and their partners had concerns about serodifference, violence, guilt and abandonment^(42)^, so alternative routes for distribution and counseling would be necessary to increase the acceptance of HIV testing among pregnant women and their partners^(42)^. Community-based testing was acceptable for MSM, but support services should be available, especially for those who have never self-tested and lack confidence in this testing method^(43)^.

HIV self-testing has been shown to be effective in increasing people’s access to confirmatory testing for infection, initiation of ART, and seeking other prevention services^(25)^. Distributing the self-test and providing guidance on other methods of combined HIV prevention, such as voluntary circumcision, PrEP and Post-Exposure Prophylaxis, makes it possible to prevent infection, reduce transmission, and provide early diagnosis and treatment, despite the difficulties in measuring patients’ connection with the healthcare service after self-test^(7,24)^.

In community self-test distributions, the article showed that there was no difference in ART initiation rates among those who tested positive^(25)^. In door-to-door distribution, lifetime HIV testing was higher when compared to testing in the healthcare service, although it did not show an effect on starting ART among those who tested positive^(27)^. Distributing self-tests among peers also did not increase adherence to ART and PrEP^(29)^. Another door-to-door distribution study found that HIV self-testing campaigns increased the initiation of ART by 27%^(24)^.

Chart 2 presents the advantages and disadvantages of the forms of distribution of the HIV self-test.

**Chart 2:** Advantages and disadvantages of strategies for implementing HIV self-testing in different populations, Fortaleza, Ceará, Brazil, 2024

Primary strategies (Self-test delivered directly to individuals at an institution or other location)		
**Distribution method**	**Advantages**	**Disadvantages**
Through apps and online ads^(7)^	Privacy and convenience, increased connection with the healthcare service	Higher financial cost
Door to door^(23-25,27)^	Awareness of self-testing^(23)^, 27% increase in antiretroviral therapy use^(24)^, improved testing coverage^(24,25)^, increased HIV testing (16.1%)^(27)^	Payment to distributors^(24)^, higher cost per self-test unit and costs per new diagnosis^(27)^
By mail^(30)^	Acceptable and viable among men who have sex with men and trans women, anonymity, and trustworthiness^(30)^	Internet access is required to access video guidance^(30)^
In pharmacies and hotspots, such as bars and nightclubs^(32)^	Increased discussion on HIV self-testing, reduction of condomless sex^(32)^	Less condom use, due to a feeling of security during unprotected sex^(32)^
At taxi stands^(36)^	Lower financial cost^(36)^	Not mentioned^(36)^
In workplaces^(36)^	Lower financial cost^(36)^	Moderate impact on preventing HIV infection^(36)^
By distribution agents in the community^(40)^	Trust between distribution agents and people receiving the self-test^(40)^	No link between people who tested positive and the healthcare service^(40)^
**Secondary strategies** **(Self-test given to individuals to be given to another person, partner or peer)**		
**Distribution method**	**Advantages**	**Disadvantages**
By opinion leaders or peer educators^(10)^	Improved self-testing reach among men who have sex with men, trust within the community, and linkage to healthcare services^(10)^	Loss of follow-up by healthcare services, difficulty in recruiting opinion leaders^(10)^
By encouraging peers on their social networks^(29)^	Reaches a high number of young people, lower financial cost when compared to direct delivery by peers^(29)^	There was no increase in the demand for Pre-Exposure Prophylaxis and antiretroviral therapy^(29)^
Direct peer distribution^(29)^	Greater post-test connection with the healthcare service^(29)^	Higher financial cost^(29)^
People with HIV on antiretroviral therapy give it to their partners^(36)^	Positive impact to prevent HIV infection^(36)^	Higher financial cost^(36)^
For sexual partnerships^(42)^	Acceptable in trusting relationships^(42)^	Fear of discovery of serodiscordance, violence and abandonment^(42)^

## DISCUSSION

In this review, it was identified that the distribution of HIV self-testing in the community was the most used, but none of the approaches in isolation had an influence on knowledge and access to HIV preventive and diagnostic measures, which shows the need for mixed strategies for distributing self-testing in different populations^(35)^.

Research has shown that distribution of self-tests in the community can benefit from monitoring and support for those who test, supervision of tests on illiterate people, and the selection of accessible leaders for distribution^(44)^. Other suggestions for enhancing community distribution include strategic support points for truck drivers, schools, colleges^(45)^ and local community centers^(46)^. However, a study of young adults in Kenya found that the preferred locations for accessing HIV self-testing were hospitals and clinics rather than community centers, schools, and universities^(47)^.

Access to testing is so important that, in a study with TW and men, 85% did not use self-testing because they did not know where it was available, i.e., due to a lack of information^(46)^. Furthermore, correct guidance on how to use the self-test is a determining factor for the success of this approach, as is the availability of visual aids that facilitate its implementation^(39)^. Studies have shown that the use of technological and audiovisual strategies, such as apps, television series and videos, promoted self-testing and helped reach populations^(31,38,39)^. However, these strategies may present some gaps, such as lack of access to the internet and other technological devices^(48,49)^.

In Brazil, for instance, a project was initiated to dispense HIV self-tests through automated dispensers and the use of applications in two large cities, with the aim of reducing costs and facilitating access^(50)^, achieving good adherence and high viability, especially in populations never tested^(16)^. Therefore, the means of distribution, human and technological resources can be decisive for the acceptability and implementation of HIV self-testing. Self-testing has had high acceptability, especially among key HIV populations^(31)^, due to its confidentiality, autonomy, and convenience^(45-47)^.

Regarding the difficulties in accepting self-testing, impacts on mental health, stigma and violence were identified^(34)^. The costs of implementing self-testing in populations were also seen as important barriers^(7)^. It is worth noting that, in Brazil, the distribution of HIV self-tests is free^(15)^, which represents an advantage for access to testing. Another concern reported in the research was the size of the test packaging to ensure easy portability^(46)^.

HIV self-testing can also be a prevention and early diagnosis measure, by improving adherence to ART and the use of PrEP^(24,35)^. When combined with PrEP, HIV self-testing has a greater chance of spreading through the social and sexual networks of those taking PrEP^(35,43)^. As an incentive for early use of antiretrovirals, HIV self-testing proved to be a determinant for greater adherence to ART and better patient connection to healthcare services, and was positively associated with disclosure of status to the partner and greater initiative to initiate ART^(24,36)^.

However, despite all the benefits of HIV self-testing, there is concern about losing opportunities to test for other sexually transmitted infections, as well as failing to offer health education activities, which shows the need to expand support platforms, with a focus on sexual health^(31,34,35)^. Furthermore, HIV self-test distribution strategies, combined with other prevention methods, have great potential, as they increase adherence to preventive measures, such as peer self-test delivery, as social interaction fosters trust, support, and appropriate language for participants^(29,31,33)^.

Secondary distribution to partners of people living with HIV on ART has a greater epidemiological impact but is less cost-effective, while distribution of HIV self-tests in workplaces is more cost-effective but has a moderate impact on HIV prevention^(36)^. Distribution in primary care is not cost-effective due to the lower number of positive tests, which becomes greater when testing key populations^(36)^. The ideal form of distribution is considered to be a combination of secondary distribution to partners of PLHIV on ART and pregnant women on treatment in primary care, workplace testing and distribution at fixed points^(36)^.

Despite these benefits, a study conducted in South Africa, which assessed 11 types of distribution, such as community distribution, found that the cost per person confirmed as positive through self-testing was higher than with standard HIV testing^(37)^. An alternative to reduce costs and human resources in the primary distribution of HIV self-testing is to perform the procedure without the presence of healthcare professionals, which takes up less of the team’s time^(26)^. In secondary distribution, video demonstration has the potential to reduce the direct time that a patient spends with counselors or distributors^(26)^.

Thus, the type of distribution varies according to the objective. If the focus is to distribute as many tests as possible, workplaces and the community are the choices; if the goal is to distribute more efficiently and at lower costs, transportation centers and workplaces are more appropriate; if the goal is to reach PLHIV partners at lower financial costs, secondary distribution through index cases at the facility and integration into existing programs for sex workers and distribution to their networks would be the most efficient options^(37)^. Considering characteristics such as distribution volume, HIV seropositivity, link with the healthcare service and costs are factors that can indicate the type of distribution to be chosen.

Given the above, HIV self-testing has proven to be an effective strategy for increasing testing rates and achieving the goals of ending the HIV pandemic. It is noted that distribution methods and methods of testing can determine whether tested individuals are linked to healthcare services, ensuring continuity of care after a positive result.

### Study limitations

A limitation of this study was the lack of research on the management of waste from HIV self-testing, which is considered potentially contaminated. A study that was not identified among the articles selected for this integrative review even highlighted the environmental damage that improper disposal of self-tests could cause^(45)^. Other limitations were the existence of few randomized clinical trials, which made it impossible to carry out a systematic review and meta-analysis as well as the reduced number of descriptors used in the search for articles in the databases.

Given these limitations, we suggest further studies on the proper management and disposal of HIV self-test waste as well as intervention and cost-effectiveness research on self-test implementation. This integrative review contributes to identifying new study topics capable of generating high-level evidence for the implementation and assessment of HIV self-tests.

### Contributions to nursing, health or public policy

The relevance of this study stands out, as it identified the main strategies for implementing HIV self-testing. These results can contribute to increasing knowledge on this topic among nurses and other healthcare professionals, as well as inform decision-making by healthcare service managers, and contribute to the development of public policies aimed at the most vulnerable.

## CONCLUSIONS

Distribution of HIV self-tests in the community and among peers was the most prevalent form of access, especially among key populations such as MSM and TW. The use of digital and technological resources facilitated access to HIV self-testing and improved individuals’ linkage to healthcare services after testing positive. Self-testing proved effective in increasing access to HIV prevention methods, such as condom use and PrEP as well as initiating ART for those who needed it. Furthermore, clinical trials are suggested to assess different strategies for implementing HIV self-testing, with an emphasis on aspects that facilitate its use, increase post-test linkage of individuals with positive results to healthcare services, and conduct cost-effectiveness studies.

## Data Availability

The research data are available within the article.
