# Supplementary material for: Strategies for implementing HIV self-testing in diverse populations: an integrative review
Source: Rev Bras Enferm. 2025 Dec 12;78(Suppl 4):e20240597. doi: 10.1590/0034-7167-2024-0597 (PMC12707773; doi:10.1590/0034-7167-2024-0597)
Supplement: Translation

# Translation: Estratégias para implementação do autoteste de HIV em diversas populações: revisão integrativa

**DOI:** 10.1590/0034-7167-2024-0597pt

**Authors:** Maiara Bezerra Dantas, Gilmara Holanda da Cunha, Régia Christina Moura Barbosa Castro, Maria Elisa Curado Gomes, Ane Kelly Lima Ramalho, Vanessa Sousa dos Santos

**Affiliations:** I Universidade Federal do Ceará. Fortaleza, Ceará, Brasil

**Keywords:** Autoteste, HIV, Diagnóstico, Promoção da Saúde, Prevenção de Doenças.

## Abstract

**Objetivos:**

identificar as estratégias para implementação do autoteste de HIV nas populações.

**Métodos:**

revisão integrativa nas bases MEDLINE/PubMed, Scopus, CINAHL, *Web of Science* e LILACS. Os critérios de inclusão foram artigos completos disponíveis eletronicamente, em português, inglês, espanhol ou francês, independente do ano de publicação. Os critérios de exclusão foram cartas ao editor, artigos de revisão e repetidos, teste rápido realizado por profissionais e autoteste doméstico, com envio para laboratório, sem resultado no local. A amostra de 24 artigos teve seu nível de evidência avaliado utilizando análise de dados descritiva.

**Resultados:**

a estratégia mais prevalente para implementação do autoteste foi a distribuição na comunidade por visita domiciliar, correios e pontos de distribuição, como bares, casas noturnas e terminais de transportes. A distribuição por pares foi expressiva entre homens que fazem sexo com homens, jovens e mulheres trans, com entrega primária direta ao indivíduo, ou secundária, em que um indivíduo entrega aos outros. As mídias sociais e tecnológicas facilitaram a captação, entrega e vinculação dos pacientes ao serviço de saúde.

**Conclusões:**

a distribuição do autoteste de HIV na comunidade foi a estratégia mais acessível, que aumentou o uso dos métodos da prevenção combinada do HIV e início da terapia antirretroviral. Os principais desafios para implementar o autoteste foram os custos financeiros e aceitação das pessoas, devido ao medo do resultado, estigma e violência pelos parceiros.

## INTRODUÇÃO

A meta 95-95-95 do *Joint United Nations Programme on HIV/AIDS* preconiza que, até 2030, 95% das pessoas vivendo com HIV (PVHIV) deverão estar diagnosticadas, 95%, em terapia antirretroviral (TARV) e 95%, em supressão viral^(1)^. O alcance dessas metas possibilitará o fim da pandemia de AIDS, além de vantagens para a saúde e economia mundial^(2)^. Para isso, a testagem é essencial, pois é o primeiro passo para vincular as PVHIV aos cuidados de saúde.

Nesse contexto, o autoteste pode colaborar para a detecção precoce do HIV e para o alcance das metas de controle global, pois é uma estratégia prática para melhorar o acesso à testagem^(3)^. O autoteste de HIV é conveniente, pois fornece privacidade e atenua as dificuldades, como o estigma e a falta de anonimato dos serviços de saúde, podendo alcançar populações inacessíveis^(4)^. Estudos mostram seu potencial para prevenção e tratamento da infecção pelo HIV^(5-7)^. No entanto, este tipo de testagem ainda apresenta alguns obstáculos, como a baixa escolaridade das pessoas que o realizam, o pensamento de que o teste não é fidedigno, por não ser realizado pelo profissional de saúde, a falta de aconselhamento prévio, o medo do resultado positivo, o vínculo fragilizado com o serviço de saúde, além da questão dos direitos humanos e liberdade, visto que o parceiro ou empregador não pode obrigar a fazer o autoteste^(8,9)^.

A forma de entrega do autoteste de HIV tem impacto no acesso à testagem e na frequência de distribuição^(10)^, mas há evidências de que é necessária a combinação de várias estratégias para sua implementação^(11-13)^. Como no Brasil é uma modalidade de testagem relativamente nova, é importante saber quais estratégias mais funcionam, qual o público e em que medida^(14,15)^, com vistas a aumentar sua eficácia e colaborar para o alcance de metas^(4)^.

No Brasil, o autoteste de HIV está em fase de implantação e ainda tem pouca visibilidade, apesar de haver diretrizes para sua distribuição, as quais são direcionadas às parcerias sexuais ou pares de pessoas que buscam a Profilaxia Pré-Exposição (PrEP), parcerias sexuais de PVHIV ou pares de pessoas testadas nos serviços de saúde, e locais de sociabilidade das populações-chave^(15)^. Sugerem-se orientações na entrega, e, em caso de necessidades dos usuários, tem-se o suporte telefônico ou pelo site^(15)^.

Desde 2019, o sistema público de saúde do Brasil iniciou a distribuição de testes em 14 cidades, no entanto, em pesquisa brasileira realizada com homens que fazem sexo com homens (HSH), 67% relataram que não sabiam que o autoteste estava disponível antes de se inscreverem no estudo^(16)^. Outra pesquisa verificou que a aceitabilidade do autoteste de HIV entre HSH foi de 47,3%^(17)^. Revisão sistemática com estudos estrangeiros mostrou que, de uma amostra de 710 participantes, 33,1% relataram que o autoteste de HIV era a modalidade preferida para testagem^(3)^.

Diante do exposto, faz-se necessário conhecer as diferentes estratégias para implementação do autoteste de HIV, para que os enfermeiros, demais membros da equipe multiprofissional de saúde e os responsáveis pelas políticas públicas possam definir o que melhor se adequa a cada população, com o intuito de permitir a acessibilidade e distribuição eficaz deste insumo.

## OBJETIVOS

Identificar as estratégias para implementação do autoteste de HIV em diversas populações.

## MÉTODOS

### Aspectos éticos

Respeitaram-se os escritos dos artigos e direitos autorais, sem modificação dos escritos encontrados em benefício do estudo ora proposto pelos autores.

### Tipo de estudo

Trata-se de revisão integrativa da literatura, conduzida em seis etapas: 1. Identificação da questão de pesquisa; 2. Busca na literatura; 3. Categorização dos estudos; 4. Avaliação dos estudos incluídos; 5. Interpretação dos resultados; 6. Apresentação da revisão^(18)^.

### Elaboração da questão de pesquisa e critérios de seleção

Elaborou-se a questão de pesquisa baseada na estratégia do acrônimo PICo (População, Interesse e Contexto): População (população geral), Interesse (estratégias de cuidado), Contexto (implementação do autoteste de HIV). Assim, formulou-se a pergunta norteadora: quais as estratégias para implementação do autoteste de HIV? Seguiram-se os itens do *Preferred Reporting Items for Systematic Reviews and Meta-Analyses* ^(19)^. Artigos completos disponíveis eletronicamente, em português, inglês, espanhol ou francês, que envolvessem a temática, independentemente do ano de publicação, para se obter o maior número de artigos possível, foram incluídos. Cartas ao editor, artigos de revisão, artigos repetidos, teste rápido realizado por profissionais de saúde e autoteste doméstico, mas com envio de material para laboratório, sem resultado no local, foram excluídos.

### Definição da amostra

Os artigos foram selecionados em cinco bases de dados, nomeadamente *Medical Literature Analysis and Retrieval System Online* (MEDLINE)/PubMed, Scopus, *Cumulative Index to Nursing and Allied Health Literature* (CINAHL), *Web of Science* e Literatura Latino-Americana e do Caribe em Ciências da Saúde (LILACS). O levantamento ocorreu de julho a dezembro de 2023, utilizando-se os descritores em português (“Infecções por HIV” e “Autoteste”) e inglês (*HIV Infections* AND *Self-Testing*) provenientes dos Descritores em Ciências da Saúde e do *Medical Subject Headings*, além do operador booleano *AND*.

As buscas foram realizadas no Portal de Periódicos da Coordenação de Aperfeiçoamento de Pessoal de Nível Superior, pela cobertura de *Internet Protocol* da Universidade Federal do Ceará, para maior alcance dos periódicos indexados e disponíveis. Para uma busca mais ampla, em cada base de dados, realizou-se um teste com a combinação dos diversos descritores para identificar o maior número possível de artigos. A Tabela 1 mostra esses cruzamentos.

**Tabela 1:** Descrição das estratégias de buscas nas bases de dados, Fortaleza, Ceará, Brasil, 2024

Bases de dados	Cruzamentos	Artigos
MEDLINE/PubMed	*“HIV Infections”* AND *Self-Testing*	3.252
Scopus	*“HIV Infections”* AND *Self-Testing*	756
CINAHL	*“HIV Infections”* AND *“Self-Testing”*	301
*Web of Science*	*“HIV Infections”* AND *Self-Testing*	138
LILACS	*“HIV Infections”* AND *Self-Testing*	42

Após as buscas nas bases de dados, os artigos foram exportados e identificados na plataforma digital *Rayyan^®^*, um *software* que facilita o processo de agrupamento dos artigos para leitura e identificação dos duplicados^(20)^. Inicialmente, foi realizada a leitura dos títulos e resumos dos artigos para avaliar se atendiam aos critérios de inclusão da revisão integrativa. Posteriormente, os artigos selecionados foram lidos na íntegra por dois revisores independentes e mascarados, baseados na pergunta norteadora do estudo e nos critérios de inclusão e exclusão. Nos casos de discordância entre os pesquisadores, um terceiro revisor esteve disponível.

### Coleta de dados

Os dados coletados foram organizados em um roteiro adaptado com informações sobre referência, ano de publicação, país de realização do estudo, objetivos, características metodológicas (tipo de estudo, população, amostra), resultados e conclusões. A seguir, fez-se uma tabela com as estratégias de cuidado para implementação do autoteste de HIV em diversas populações. Os níveis de evidência dos artigos foram classificados em: I. Evidências provenientes de revisão sistemática ou metanálise de ensaios clínicos randomizados controlados ou diretrizes clínicas baseadas em revisões sistemáticas de ensaios clínicos randomizados controlados; II. Evidências de pelo menos um ensaio clínico randomizado controlado; III. Evidências de ensaios clínicos sem randomização; IV. Evidências de estudos de coorte e caso-controle; V. Evidências de revisão sistemática de estudos descritivos e qualitativos; VI. Evidências de um estudo descritivo ou qualitativo; VII. Evidências da opinião de autoridades ou relatório de comitês de especialistas^(21)^. A avaliação foi realizada por pares de modo aberto, na qual participaram três autores do estudo, para os casos em que não houvesse consenso quanto à definição dos níveis de evidência.

## RESULTADOS

Um total de 4.489 artigos foram selecionados, e 24 compuseram o estudo. O ano de publicação dos artigos variou entre 2017 e 2023. Quanto aos níveis de evidência, oito eram nível II^(22-29)^, um, nível III^(30)^, três, nível IV^(31-33)^ e 12, nível VI^(7,10,34-43)^. A Figura 1 mostra o fluxograma de seleção.

**Figura 1:** Fluxograma dos artigos encontrados e selecionados nas bases de dados, Fortaleza, Ceará, Brasil, 2024

As estratégias para distribuição do autoteste mais prevalentes nos estudos foram distribuição na comunidade^(7,23-25,27,28,30,32,36,37,40,41)^ e distribuição por pares^(10,26,29,31,33,36,42)^. As populações mais participantes foram de HSH^(7,10,30,31,34,43)^, jovens^(29,38-41)^ e mulheres trans (MT)^(30,34)^. A síntese descritiva dos estudos é apresentada no Quadro 1.

**Quadro 1:** Estratégias para implementação do autoteste de HIV em diversas populações, Fortaleza, Ceará, Brasil, 2024

Autor/ano/ país de realização	Objetivos	Método	População	Resultados e conclusões	Nível de evidência^(21)^
Edelstein *et al*. (2020) Estados Unidos^(7)^	Descrever o *Home Test Giveaway 2015-2018*, seu processo de intervenção e resultados.	Estudo transversal *online*	n= 7.935	Na entrega de forma *online* e em domicílio, os participantes recomendaram o autoteste de HIV pela privacidade e conveniência, relatando seus resultados, e os com teste positivo tiveram alto vínculo com o serviço de saúde.	VI
Sekoni *et al*. (2022) Nigéria^(10)^	Avaliar a entrega do autoteste de HIV aos HSH por líderes de opinião.	Estudo qualitativo	12 grupos distribuidores e 20 HSH	Líderes de opinião descreveram que a entrega do autoteste de HIV foi bem-sucedida e que a confiança foi um determinante. Foram vistos pelos HSH como fontes seguras de educação interpessoal, com destaque para o treinamento sobre autoteste. Oferta de suporte, gestão de pessoas, paciência e perseverança são características importantes dos líderes, sendo um recurso para promover o autoteste entre os HSH.	VI
Sande *et al*. (2018) Malawi^(22)^	Identificar e quantificar os custos dos clientes do serviço para autoteste de HIV em comunidades rurais no Malawi.	Ensaio clínico randomizado por *cluster* Pesquisa em domicílio e entrevista retrospectiva	n=749	Para aqueles que tiveram custo, a média foi de US$ 2,06. Idade, local, tempo para adquirir o autoteste, visita à instituição e local de residência influenciam os custos. Dos participantes, 23% não tiverem custo para adquirir autoteste. Participantes que testam na própria comunidade têm 61% menos custos financeiros, mas a faixa etária mais velha tem maiores custos.	II
Neuman *et al*. (2021) Zâmbia^(23)^	Avaliar o impacto da distribuição do autoteste de HIV baseado na comunidade em serviços de testagem.	Ensaio clínico randomizado por *cluster*	n=5.005	Quando comparado ao serviço de testagem padrão em unidades de saúde (controle) e a distribuição na comunidade (intervenção), apenas 58,0% dos entrevistados no braço intervenção conheciam o autoteste, comparados com 28,3% no controle. A distribuição baseada na comunidade não aumentou o uso do autoteste, mas melhorou a conscientização sobre testagem.	II
Sibanda *et al*. (2021) Zimbábue^(24)^	Avaliar o efeito do incentivo ao vínculo com o paciente após autoteste de HIV e a tendência temporal para início da TARV.	Ensaio clínico randomizado por *cluster*	n=7.146	Não houve evidência de benefício em fornecer aos distribuidores de *kits* do autoteste de HIV um incentivo de vinculação, além de um salário fixo. A distribuição porta a porta de *kits* para autoteste de HIV na comunidade é viável e melhorou a cobertura da testagem.	II
Sibanda *et al*. (2021) Zimbábue^(25)^	Comparar um modelo para autoteste de HIV baseado na comunidade versus modelo estabelecido na zona rural do Zimbábue.	Ensaio clínico randomizado por *cluster*	n=11.150, Grupo 1: liderado pela comunidade (n=5.683) Grupo 2: distribuidor pago (n=5.467)	Captação para autoteste de HIV foi menor no braço da comunidade (21,6%), comparada ao braço distribuidor pago (27,5%). O vínculo pós-teste com o serviço de saúde foi maior no braço liderado pela comunidade. Os custos financeiros no braço distribuidor pago foram menores. A intervenção baseada na comunidade é promissora. Os custos tendem a reduzir com o tempo e maior implementação.	II
Sande *et al*. (2021) Malawi, África do Sul, Zâmbia e Zimbábue^(26)^	Avaliar os custos de adicionar o autoteste de HIV aos testes de HIV padrão em quatro países.	Ensaio clínico randomizado por *cluster*	n=41.720	Os conselheiros gastaram uma média de 20-44% do tempo em atividades de autoteste para HIV. O custo em instalações públicas variou de US$ 4,27 a US$ 13,42 por *kit* distribuído nos países. A distribuição primária variou de US$ 4,27 a US$ 9,24, enquanto a secundária variou de US$ 6,46 a US$ 13,42.	II
Indravudh *et al*. (2021) Malawi^(27)^	Avaliar a eficácia e segurança da distribuição porta a porta de *kits* de autoteste de HIV na zona rural do Malawi.	Ensaio clínico randomizado	n=5.490	A distribuição porta a porta do autoteste de HIV por agentes de distribuição na comunidade aumentou a testagem para HIV (16,1%) e a testagem ao longo da vida (6,3%), sobretudo em adolescentes de 16 a 19 anos homens.	II
Indravudh *et al*. (2021) Malawi^(28)^	Avaliar os custos e efeitos da entrega de autoteste para HIV liderada pela comunidade.	Ensaio pragmático randomizado por *cluster*	n=7.880	Na entrega conduzida pela comunidade, observou-se um baixo custo unitário adicional. O autoteste para HIV, liderado pela comunidade junto ao padrão de atendimento, não seria custo-efetivo em locais com baixa prevalência de HIV.	II
Shahmanes *et al*. (2021) África do Sul^(29)^	Investigar dois modelos de distribuição do autoteste de HIV por pares para prevenção do HIV em jovens.	Ensaio clínico randomizado por *cluster*	n=24 pares	A distribuição do autoteste de HIV entre os pares alcançou grande número de jovens. Já a incentivada por redes sociais também atingiu grande número de jovens, mas resultou em menor vínculo com o serviço de saúde.	II
Rosadiño *et al*. (2023) Filipinas^(30)^	Avaliar a viabilidade do modelo de distribuição de autoteste de HIV na comunidade e avaliar sua aceitabilidade entre HSH e MT.	Estudo quase-experimental, com aplicação de questionário	n=4.205	A implementação do autoteste com distribuição na comunidade, realizada com a ajuda de assistente virtual e envio do teste por correios, foi viável. Materiais instrucionais e vídeos podem ajudar a garantir o uso correto do autoteste de HIV.	III
John *et al*. (2020) Estados Unidos^(31)^	Determinar a disposição de homens *gays* e bissexuais para ofertar autoteste de HIV e TARV ao parceiro, e se envolver na notificação, com uso do aplicativo.	Estudo de coorte	n=786	Na entrega feita aos parceiros sexuais, os que relataram sexo anal sem preservativo com parceiro casual tiveram 3,21 vezes mais chances de estarem dispostos a ofertar o autoteste de HIV e tratamento para outras infecções sexualmente transmissíveis aos parceiros. Nos homens solteiros, 93,8% dos que relataram sexo anal recente sem preservativo com parceiro casual estavam dispostos a ofertar o autoteste de HIV e tratamento para outras infecções sexualmente transmissíveis ao parceiro.	IV
Driver *et al*. (2023) Quênia^(32)^	Avaliar se a conclusão do autoteste está associada a mudanças no comportamento sexual e percepção de risco.	Estudo de coorte com aplicação de questionário	n=221	Os modelos de distribuição comunitária do autoteste foram distribuição domiciliar, em farmácias, divulgação em bares e casas noturnas. Após o autoteste de HIV, os jovens discutiram mais sobre testagem de forma geral. Aqueles que receberam o autoteste em farmácia e domicílio reduziram atos sexuais sem preservativo. Oferecer o autoteste à população pode motivar a falar sobre testagem com os parceiros.	IV
Gosset *et al*. (2023) França^(33)^	Avaliar a proporção e características de pessoas que distribuíram e que receberam o autoteste.	Estudo de intervenção prospectivo, com questionários	n=682	Autotestes foram entregues a pacientes para entregar às pessoas de suas redes sociais. Os que distribuíram ao menos um autoteste tiveram mais chances de uso anterior do autoteste. Os mais velhos tiveram menos probabilidade de distribuir o autoteste. A sensibilização prévia e capacitação para uso melhoraram a distribuição.	IV
Wirtz *et al*. (2017) Mianmar^(34)^	Investigar como o autoteste de HIV pode beneficiar HSH e MT nos cuidados pós-teste.	Pesquisa qualitativa, com grupo focal	n=35	A educação comunitária e mídia de massa para informar os HSH e MT sobre o autoteste de HIV são importantes para garantir o acesso dos HSH e MT às instruções sobre uso do autoteste.	VI
Durosinmi-Etti *et al*. (2021) Nigéria^(35)^	Entender facilidades, barreiras e necessidades de comunicação para populações-chave na oferta de PrEP e autoteste de HIV na Nigéria.	Estudo transversal, com entrevistas e grupo focal	n=1.169	Facilitadores do autoteste foram interação presencial, sessões de educação por pares, mídias sociais, oferta do autoteste quando a pessoa adquire produto semelhante, redes comunitárias, mensagens em diversos idiomas. A interação presencial foi a mais conscientizadora sobre autoteste de HIV (85% MTS; 68% HSH), seguida das mídias sociais (9,4% MTS; 31,6% HSH). A influência dos pares é essencial para propagar mensagens sobre autoteste de HIV.	VI
Jamieson *et al*. (2021) África do Sul^(36)^	Avaliar o impacto de seis modalidades de distribuição de *kits* para autoteste de HIV na África do Sul.	Estudo transversal por telefone e avaliação econômica	n=40.834	A distribuição secundária a parceiros de pacientes em TARV tem impacto epidemiológico para evitar novas infecções e melhorar a sobrevida, mas é uma estratégia pouco rentável. O ponto de táxi e distribuição no local de trabalho são estratégias mais econômicas.	VI
Matsimela *et al*. (2021) África do Sul^(37)^	Analisar o custo, uso e relação com os cuidados posteriores de 11 modelos de distribuição de *kits* para autoteste de HIV.	Estudo transversal por meio de pesquisa telefônica	n=40.834	Custos médios por *kit* do autoteste de HIV variaram de US$ 4,87 (modelo de trabalhadora do sexo) a US$ 18,07 (modelo de integração móvel). Modelo de profissionais do sexo, terminais de transporte e local de trabalho são mais eficientes e menos onerosos. Se o objetivo é distribuir *kits* de forma barata e que encontre mais pessoas HIV+, a distribuição secundária por caso índice nas instalações e por profissionais do sexo é mais eficiente.	VI
Nwaozuru *et al*. (2021) Nigéria^(38)^	Examinar a participação dos jovens em maratona para criar serviços de saúde amigos dos jovens na Nigéria.	Estudo descritivo	n=48 jovens	As três abordagens finalistas eram voltadas para descentralizar os serviços aos jovens por dispositivos e tendas móveis, tecnologias de saúde ou serviços de apoio aos pares. A estratégia vencedora foi a criação de aplicativo móvel para distribuição e solicitação do autoteste de HIV.	VI
Birdthistle *et al*. (2022) África do Sul^(39)^	Avaliar o impacto da série *MTV Shuga Down South* na prevenção do HIV, aceitação e conscientização do autoteste de HIV e PrEP^§^ em jovens na África do Sul.	Estudo misto, pesquisa *online*	n=3.431	Aqueles que assistiram à série tiveram maior conhecimento da sorologia anti-HIV (71%), usaram mais autoteste de HIV (29%) ou tiveram alto interesse em usá-lo (83%), acessaram com mais frequência os serviços de testagem, e tiveram mais conhecimentos.	VI
Harrison *et al*. (2022) Malawi^(40)^	Explorar barreiras e facilitadores do vínculo com serviço de saúde para prevenção e cuidados do HIV após autoteste em jovens.	Estudo transversal, com entrevista e grupos focais	n=41	A comunicação sobre benefícios do teste e o encaminhamento pós-teste foram ruins, segundo os jovens. Eles valorizam o encorajamento daqueles em quem confiavam, experiências positivas de tratamento de outros e “força de espírito”. Agentes de distribuição na comunidade promoveram relações de confiança e maior compreensão dos fatores que impediram os jovens de se vincularem após o autoteste.	VI
Aizobu *et al*. (2023) Nigéria^(41)^	Identificar os facilitadores e barreiras para realizar o autoteste usando a metodologia mapa de jornada.	Estudo exploratório, com entrevista e grupo focal	n=80	Os jovens preferiam que a farmácia entregasse o autoteste de HIV em domicílio, pois tinham medo de serem vistos na farmácia. Apesar de preferirem esse modelo de entrega, reconheciam a falta do aconselhamento que tem no serviço de saúde público, mas não na farmácia.	VI
Bulterys *et al*. (2023) Uganda^(42)^	Avaliar as perspectivas de grávidas e parceiros sobre a aceitabilidade, barreiras, facilitadores e estratégias para autoteste de HIV.	Estudo exploratório, com entrevista e grupo focal	n=122	A distribuição secundária foi aceitável, mas os casais preferiam testar de forma individual. Ambos hesitaram em testar na presença do parceiro. As gestantes relataram a importância de receber orientações sobre o uso e interpretação do autoteste, além de estratégias para abordar o parceiro.	VI
Kwan *et al*. (2023) China^(43)^	Avaliar a cascata de implementação de abordagem do autoteste de HIV baseada em redes sociais com HSH.	Estudo transversal, com aplicação de questionário	n=442	Usar as redes sociais de HSH para recrutar outros homens é uma abordagem viável, mas precisa de pares motivados. Ter usuários confiantes para recrutamento pode ser benéfico, pois eles têm experiência, conhecimento e relacionamento com os pares.	VI

*HSH - homens que fazem sexo com outros homens; TARV - terapia antirretroviral; MT - mulher trans; PrEP - Profilaxia Pré-Exposição; MTS - mulheres trabalhadoras do sexo.*

A distribuição do autoteste de HIV pode ocorrer de forma primária, em que o autoteste é entregue diretamente ao indivíduo, dentro de uma instituição ou outro local, ou de forma secundária, na qual é entregue a um indivíduo, para que este entregue a outra pessoa, como ao seu parceiro sexual^(36)^ ou aos seus pares^(31)^.

A distribuição na comunidade foi a mais comum^(7,23-25,27,28,30,32,36,37,40,41)^. Esta pode acontecer de inúmeras formas, com a ajuda de pessoas chamadas distribuidores comunitários, que entregam os autotestes por visita domiciliar^(23,32,41)^, por meio dos correios^(7,30)^ e em pontos de distribuição em locais frequentados pela população a ser alcançada, como bares e casas noturnas^(32,36)^. Em terminais de transportes (praças de táxis e estações ferroviárias com elevado tráfego de pedestres), os autotestes são oferecidos aos passageiros, motoristas de táxi e vendedores ambulantes.

Nesses terminais, os distribuidores fazem a demonstração do uso do autoteste, oferecendo-os aos interessados para uso privado fora do local^(36,37)^. Por distribuição porta a porta, as pessoas vão até o domicílio oferecer e entregar o autoteste^(24,25,27)^. A distribuição também pode acontecer com e sem incentivo financeiro, em que os distribuidores recebem um pagamento para realizar esta atividade^(24,25)^.

Observou-se a expressiva distribuição pelos pares nos estudos avaliados, ocorrendo pelos parceiros sexuais^(31)^, por gestantes aos seus parceiros sexuais durante o pré-natal^(26,42)^ e por pessoas com HIV aos seus parceiros^(26,36)^, entregando às pessoas de suas redes sociais^(29,33)^. Um dos estudos abordou a participação dos líderes de opinião, que eram pessoas respeitadas na comunidade, para distribuir o autoteste de HIV entre os HSH, bem como estimular que estes buscassem cuidados em saúde^(10)^.

Para distribuição dos autotestes, alguns pesquisadores utilizaram mídias sociais para facilitar a captação, entrega e vinculação dos pacientes ao serviço de saúde após a testagem. Foram utilizados aplicativos móveis^(31,38)^, como de namoro^(7)^ ou para notificação de parceiros positivos para HIV^(31)^, *WhatsApp^®^* ^(10)^, mídias, como séries de TV^(39)^, e anúncios *online* em mídias digitais^(7)^.

Quanto à aceitabilidade do autoteste de HIV, os participantes que realizaram o autoteste relataram altos níveis de acompanhamento em saúde quando testaram positivo e que recomendariam esta forma de testagem, devido à privacidade e conveniência domiciliar^(7,34)^. Já em relação aos aspectos que dificultam a aceitabilidade do autoteste de HIV, estão a ausência de aconselhamento pré-teste e pós-teste, pois podem impactar negativamente a saúde mental, com menor possibilidade de revelar o *status* de HIV+, notificação e vigilância dos casos^(34)^.

Em estudo com homens *gays* e bissexuais, a maioria que foi diagnosticada por meio do autoteste de HIV considerou aceitável disponibilizar ao parceiro o tratamento para outras infecções sexualmente transmissíveis^(31)^. Já as mulheres grávidas e seus parceiros preferiram fazer o autoteste de forma independente, ou seja, que este não fosse ofertado pela própria parceria^(42)^.

Embora aceitável para casais em relações de confiança e compreensão mútua, as gestantes e seus parceiros tiveram a preocupação quanto à sorodiferença, violência, culpa e abandono^(42)^, de modo que seriam necessárias vias alternativas para distribuição e aconselhamento para aumentar a aceitação da testagem anti-HIV entre gestantes e seus parceiros^(42)^. Testes baseados na comunidade foram aceitáveis para HSH, mas devem existir serviços de apoio, sobretudo para os que nunca fizeram o autoteste e não possuem confiança nessa modalidade de testagem^(43)^.

O autoteste de HIV mostrou-se efetivo para aumentar o acesso das pessoas aos testes confirmatórios da infecção, início da TARV e busca de outros serviços de prevenção^(25)^. Distribuir o autoteste e orientar sobre outros métodos da prevenção combinada do HIV, como a circuncisão voluntária, PrEP e Profilaxia Pós-Exposição, possibilita prevenir a infecção, reduzir a transmissão, além de fazer o diagnóstico e tratamento precoce, apesar das dificuldades em mensurar o vínculo do paciente com o serviço de saúde após o autoteste^(7,24)^.

Em distribuições do autoteste na comunidade, artigo mostrou que não houve diferença nas taxas de início da TARV entre os que testaram positivo^(25)^. Na distribuição porta a porta, a testagem para HIV ao longo da vida foi maior quando comparada à testagem no serviço de saúde, apesar de não mostrar efeito para iniciar a TARV entre os que testaram positivo^(27)^. A distribuição de autoteste entre os pares também não aumentou a adesão à TARV e à PrEP^(29)^. Já outro estudo de distribuição porta a porta identificou que campanhas para autoteste de HIV aumentaram em 27% o início da TARV^(24)^.

Apresenta-se o Quadro 2 com as vantagens e desvantagens das formas de distribuição do autoteste para HIV.

**Quadro 2:** Vantagens e desvantagens das estratégias para implementação do autoteste de HIV em diversas populações, Fortaleza, Ceará, Brasil, 2024

Estratégias primárias (Autoteste entregue diretamente ao indivíduo em uma instituição ou outro local)		
**Forma de distribuição**	**Vantagens**	**Desvantagens**
Por meio de aplicativos e anúncios *online* ^(7)^	Privacidade e conveniência, aumento do vínculo com o serviço de saúde	Maior custo financeiro
Porta a porta^(23-25,27)^	Conscientização sobre o uso do autoteste^(23)^, aumento de 27% no uso da terapia antirretroviral^(24)^, melhora da cobertura de testagem^(24,25)^, aumento da testagem para HIV (16,1%)^(27)^	Pagamento aos distribuidores^(24)^, maior custo por unidade de autoteste e custos por novo diagnóstico^(27)^
Pelos correios^(30)^	Aceitável e viável entre homens que fazem sexo com homens e mulheres trans, anonimato, e confiabilidade^(30)^	Necessidade de acesso à internet para obter as orientações por vídeo^(30)^
Em farmácias e pontos críticos, como bares e casas noturnas^(32)^	Maior discussão sobre o autoteste para HIV, redução do sexo sem preservativo^(32)^	Menor uso do preservativo, por sensação de segurança no sexo desprotegido^(32)^
Em pontos de táxi^(36)^	Menor custo financeiro^(36)^	Não referida^(36)^
Em locais de trabalho^(36)^	Menor custo financeiro^(36)^	Impacto moderado em prevenir a infecção pelo HIV^(36)^
Por agentes de distribuição na comunidade^(40)^	Confiança entre os agentes de distribuição e as pessoas que recebiam o autoteste^(40)^	Não vinculação das pessoas que testavam positivo com o serviço de saúde^(40)^
**Estratégias secundárias** **(Autoteste entregue ao indivíduo, para que fosse entregue a outra pessoa, parceiro ou pares)**		
**Forma de distribuição**	**Vantagens**	**Desvantagens**
Por líderes de opinião ou educadores de pares^(10)^	Melhor alcance do autoteste em homens que fazem sexo com homens, confiança com a comunidade, vinculação ao serviço de saúde^(10)^	Perda de acompanhamento pelos serviços de saúde, dificuldade no recrutamento de líderes de opinião^(10)^
Por pares incentivadores nas suas redes sociais^(29)^	Atinge alto número de jovens, menor custo financeiro quando comparado à entrega direta pelos pares^(29)^	Não aumentou a demanda de Profilaxia Pré-Exposição e terapia antirretroviral^(29)^
Distribuição direta por pares^(29)^	Maior vínculo pós-teste com o serviço de saúde^(29)^	Maior custo financeiro^(29)^
A pessoas com HIV em terapia antirretroviral entrega aos seus parceiros^(36)^	Impacto positivo para prevenir a infecção pelo HIV^(36)^	Maior custo financeiro^(36)^
Pelas parcerias sexuais^(42)^	Aceitável em relações confiantes^(42)^	Medo da descoberta da sorodiscordância, violência e abandono^(42)^

## DISCUSSÃO

Nesta revisão, identificou-se que a distribuição do autoteste de HIV na comunidade foi a mais utilizada, mas nenhuma das abordagens de maneira isolada teve influência no conhecimento e acesso às medidas preventivas e diagnósticas do HIV, o que mostra a necessidade de estratégias mistas para distribuição do autoteste em diferentes populações^(35)^.

Pesquisa apontou que a distribuição do autoteste na comunidade pode ser beneficiada com o acompanhamento e apoio a quem testa, supervisão dos testes em analfabetos, além da escolha de líderes acessíveis para distribuição^(44)^. Outras sugestões para potencializar a distribuição na comunidade incluem pontos de apoio estratégicos para motoristas de caminhão, escolas, faculdades^(45)^ e centros comunitários locais^(46)^. Porém, estudo com adultos jovens no Quênia observou que os locais preferidos para acessar o autoteste de HIV foram hospitais e clínicas, em detrimento aos centros comunitários, escolas e universidades^(47)^.

O acesso à testagem é tão importante que, em estudo com MT e homens, 85% não utilizaram o autoteste por não saberem onde estavam disponíveis, ou seja, pela falta de informação^(46)^. Além disso, a orientação correta de uso do autoteste é um fator determinante para o sucesso dessa abordagem, assim como a disponibilidade de meios visuais que facilitem a realização^(39)^. Estudos mostraram que o uso de estratégias tecnológicas e audiovisuais, tais como aplicativos, séries televisivas e vídeos, promoveu o autoteste e colaborou para o alcance das populações^(31,38,39)^. No entanto, essas estratégias podem apresentar algumas lacunas, como a falta de acesso à internet e demais dispositivos tecnológicos^(48,49)^.

No Brasil, por exemplo, foi iniciado um projeto para dispensação de autotestes de HIV por meio de dispensadores automatizados e uso de aplicativos em duas grandes cidades, com o objetivo de reduzir os custos e facilitar o acesso^(50)^, obtendo-se uma boa adesão e alta viabilidade, especialmente em populações nunca testadas^(16)^. Diante disso, os meios de distribuição, os recursos humanos e tecnológicos podem ser determinantes para a aceitabilidade e realização do autoteste de HIV. O autoteste teve alta aceitabilidade, especialmente em populações-chave para HIV^(31)^, devido à confidencialidade, autonomia e conveniência^(45-47)^.

No que diz respeito às dificuldades para aceitação do autoteste, foram identificados os impactos na saúde mental, estigma e violência^(34)^. Os custos para implementação do autoteste nas populações também foram vistos como importantes barreiras^(7)^. Vale ressaltar que, no Brasil, a distribuição do autoteste de HIV é gratuita^(15)^, o que representa uma vantagem para o acesso aos testes. Outra preocupação relatada em pesquisa foi o tamanho das embalagens dos testes para que tivessem fácil portabilidade^(46)^.

O autoteste de HIV pode ser também uma medida de prevenção e diagnóstico precoce, ao melhorar a adesão à TARV e o uso da PrEP^(24,35)^. Quando em associação com a PrEP, o autoteste de HIV emerge com maior possibilidade de disseminação nas redes sociais e sexuais daqueles que recebem a PrEP^(35,43)^. Como incentivador para o uso precoce dos antirretrovirais, o autoteste de HIV mostrou-se como um determinante para maior adesão à TARV e melhor vinculação dos pacientes ao serviço de saúde, e foi positivamente associado à revelação do *status* ao parceiro e maior iniciativa para iniciar a TARV^(24,36)^.

No entanto, apesar de todos os benefícios do autoteste de HIV, há o receio da perda de oportunidades para testagem de outras infecções sexualmente transmissíveis, assim como deixar de ofertar atividades de educação em saúde, o que mostra a necessidade de ampliação das plataformas de apoio, com foco na saúde sexual^(31,34,35)^. Ademais, as estratégias de distribuição do autoteste de HIV, associadas a outros meios de prevenção, têm grande potencial, pois aumentam a adesão às medidas preventivas, como na entrega do autoteste por pares, já que a interação social propicia a confiança, apoio e linguagem adequada aos participantes^(29,31,33)^.

A distribuição secundária para parceiros de PVHIV em TARV tem maior impacto epidemiológico, porém é menos custo-efetiva, enquanto a distribuição do autoteste de HIV em locais de trabalho é mais econômica, porém com impacto moderado na prevenção do HIV^(36)^. A distribuição na atenção primária não é custo-efetiva, devido a menor quantidade de testes positivos, o que se torna maior quando se testam as populações-chave^(36)^. Considera-se que a forma de distribuição ideal seja uma combinação entre distribuição secundária para parceiros de PVHIV em TARV e mulheres grávidas em tratamento na atenção primária, os testes no local de trabalho e a distribuição em pontos fixos^(36)^.

Apesar desses benefícios, estudo realizado na África do Sul, que avaliou 11 tipos de distribuição, como a distribuição comunitária, observou que o custo por pessoa confirmada como positiva por meio do autoteste foi maior que no teste padrão de HIV^(37)^. Uma alternativa para reduzir os custos e recursos humanos na distribuição primária do autoteste de HIV é realizar o procedimento sem a presença dos profissionais de saúde, o que compromete menos o tempo da equipe^(26)^. Na distribuição secundária, a demonstração por meio de vídeos tem o potencial de reduzir o tempo direto que o paciente demanda com os conselheiros ou distribuidores^(26)^.

Assim, o tipo de distribuição varia de acordo com o objetivo. Se o foco é distribuir o maior número de testes possível, as escolhas são os locais de trabalho e na comunidade; se é distribuir de forma mais eficiente e com menos custos, os centros de transporte e locais de trabalho são mais indicados; se é alcançar as parcerias das PVHIV com menor custos financeiros, a distribuição secundária por meio de casos índice na unidade e a integração em programas existentes para profissionais do sexo e distribuição para suas redes seriam as escolhas mais eficientes^(37)^. Considerar as características como o volume da distribuição, soropositividade para HIV, vínculo com o serviço de saúde e custos são fatores que podem apontar o tipo de distribuição a ser escolhida.

Diante do exposto, o autoteste de HIV se revela como uma estratégia efetiva para elevar as taxas de testagem e alcançar as metas para acabar com a pandemia de infecção pelo HIV. Observa-se que os modos de distribuição e meios para realização são capazes de determinar a vinculação dos indivíduos testados aos serviços de cuidados em saúde, de modo a garantir a continuidade da assistência após o resultado positivo.

### Limitações do estudo

Como limitação deste estudo, teve-se a ausência de pesquisas sobre o gerenciamento dos resíduos provenientes da realização do autoteste de HIV, os quais são considerados potencialmente contaminados. Estudo que não foi identificado entre os artigos selecionados nesta revisão integrativa chegou a apontar os danos ambientais que o descarte incorreto do autoteste poderia causar^(45)^. Outras limitações foram a existência de poucos ensaios clínicos randomizados, o que impossibilitou a realização de revisão sistemática e metanálise, assim como o reduzido número de descritores utilizados na busca dos artigos nas bases de dados.

Diante das limitações, sugerem-se a realização de estudos sobre o manejo e o descarte correto dos resíduos de autotestes para HIV, assim como pesquisas de intervenção e custo-efetividade sobre a implementação do autoteste. A presente revisão integrativa contribui para apontar novas temáticas de estudos capazes de gerar evidências de alto nível para implementação e avaliação dos autotestes de HIV.

### Contribuições para as áreas da enfermagem, saúde ou políticas públicas

Destaca-se a relevância deste estudo, o qual identificou as principais estratégias para implementação do autoteste de HIV. Esses resultados podem contribuir para aumentar o conhecimento acerca da temática pela enfermagem e demais profissionais de saúde, além de embasar a tomada de decisão pelos gestores dos serviços de saúde e colaborar para o desenvolvimento das políticas públicas voltadas aos mais vulneráveis.

## CONCLUSÕES

A distribuição do autoteste de HIV na comunidade e por pares foi a forma mais prevalente de acesso, especialmente em populações-chave como HSH e MT. O uso de recursos digitais e tecnológicos facilitou o acesso ao autoteste de HIV, assim como melhorou a vinculação do indivíduo ao serviço de saúde após a testagem positiva. O autoteste mostrou-se eficaz para o acesso aos meios preventivos da infecção pelo HIV, tais como o uso do preservativo e da PrEP, assim como o início da TARV para aqueles que precisavam. Ademais, sugere-se o desenvolvimento de ensaios clínicos para avaliar as diferentes estratégias de implementação do autoteste de HIV, com ênfase nos aspectos que facilitem sua utilização, aumentem o vínculo pós-teste dos indivíduos com resultado positivo aos serviços de saúde, assim como a realização de pesquisas de custo-efetividade.

## Data Availability

Os dados da pesquisa estão disponíveis no corpo do artigo.
